# Social Security Satisfaction and People’s Subjective Wellbeing in China: The Serial Mediation Effect of Social Fairness and Social Trust

**DOI:** 10.3389/fpsyg.2022.855530

**Published:** 2022-04-04

**Authors:** Na Li, Mang He

**Affiliations:** School of Public Policy and Administration, Chongqing University, Chongqing, China

**Keywords:** social security satisfaction, social fairness, social trust, subjective wellbeing, mediation

## Abstract

**Objective:**

To test the relationship between social security satisfaction, social fairness, social trust, and people’s subjective wellbeing (SWB) in China and the serial mediation effect in this study.

**Methods:**

We utilized the data (*N* = 7,978) from Chinese Social Survey (CSS) in 2017 and 2019, involving 31 provinces across the country. There were 5,398 samples in 2017CSS and 2,580 samples in 2019CSS selected by the research objectives. There were 4,269 women and 3,709 men with the average age of participants being 43 (SD = 14.41).

**Results:**

The results showed that the actual status of social security satisfaction, social fairness and trust, and SWB were greater than the theoretical status overall. Social security satisfaction [*β* = 0.454, *p* < 0.001, 95% CI = (0.377, 0.423)], social fairness [*β* = 0.065, *p* < 0.001, 95% CI = (−0.039, 0.124)], and social trust [*β* = 0.108, *p* < 0.001, 95% CI = (0.237, 0.397)] positively influenced people’s SWB, respectively. Social fairness had a positive effect on social trust (*β* = 0.298, *p* < 0.001). Social fairness and social trust partly mediated the relationship between social security satisfaction and SWB, respectively. Social security satisfaction indirectly influenced SWB through the serial effect of social fairness and social trust. The total effect of SWB explained is 47% in the serial mediation model.

**Conclusion:**

Satisfactory social security is likely to cause a high level of people’s SWB, social fairness, and social trust. It is beneficial to form a virtuous circle in society. Allowing people to obtain satisfactory social security is conducive to social equity, promoting social trust, and improving people’s SWB.

## Introduction

Subjective wellbeing (SWB) is a subjective evaluation of an individual’s attitude to life, reflected by happiness or life satisfaction, which is a crucial conception developed by Diener and researchers has been accepted worldwide (Larsen et al., 1985; Diener et al., 1997; Pavot and Diener, 2008). Recent studies have analyzed the trends of individual SWB at the global level. Although the SWB level is various in different countries, the factors involved are consistent (Jebb et al., 2020). To some extent, social factors have a greater effect on SWB than sociodemographic factors (Siedlecki et al., 2013; Appau et al., 2019). After all, social problems involving the absence of health care, unemployment, and income inequality are intimately related to daily life, while citizens’ SWB is a critical index for assessing the social status as well as a possible pursuit of government or public policies (Staveren et al., 2013; Suriyanrattakorn and Chang, 2021).

In the past 30 years, especially, China’s economy has developed rapidly with the urbanization rate approaching 60% (Chen et al., 2019a). People placed higher demands on the quality of life and public services. The government exerts effective social management and services to ensure the speed and quality of social development and improve people’s life satisfaction, which also facilitates building the government’s credibility (Liu et al., 2020). In this way, the report of the 19th National Congress of the Communist Party of China pointed that the government should shoulder the responsibility of enhancing public services and meeting people’s ever-growing needs for a better life. Promoting social fairness and justice will make people happier. The “14th Five-Year Plan” in China outlined that social security system is organically integrated with public services, rights and interests of vulnerable groups, employment, income distribution, and grass-roots social governance, giving it a vital role in strengthening people’s livelihood and wellbeing. It showed that social security is indispensable to uphold social fairness and justice and promote happiness in life.

It is worth noting that there are still gaps in empirical research in the Chinese context through literature review. First, fairness and trust have been correlated in previous research, but no one has discussed their effects between social security satisfaction and SWB. Second, as the elements of the social system, these variables may be related to a certain extent according to societal theories, but what the relationship is in the Chinese context is unknown. Third, although prior studies have developed a simple mediation model, the multiplex model of these factors is also urgently needed. Hence, this research would comprehensively analyze the relationship between social security satisfaction and SWB from the people’s view in China and check the effect of social fairness and social trust in this relationship. We would like to answer the following questions. How does social security satisfaction influence people’s SWB in China? What is the role of social fairness and social trust between social security satisfaction and SWB? Whether social fairness and social trust play a serial-mediated role? The research findings are hoped to contribute to the theoretical and practical development.

## Literature Review and Hypothesis

### Social Security Satisfaction and People’s SWB

Social security satisfaction in this study refers to a subjective evaluation of social security. Social security in China is the state or the government through the redistribution of national income to achieve the goal of meeting citizens’ basic living needs. The social security system mainly comprises social insurance, social assistance, preferential social treatment, and social welfare in various fields. The lowest-level need of human beings is the survival need in Maslow’s need hierarchy theory. They do not purse higher-level needs such as self-worth until the survival is satisfied (Maslow, 1943). A dominant role of social security is to support disadvantaged groups so that they can receive material security, such as Pension Insurance (Wahba and Bridwell, 1976; Ng et al., 2017), Employment Security (Tang et al., 2018), Medical Security (Mao and Han, 2018), Housing Security (Winston, 2017), urban and rural subsistence allowances (Ding, 2017). Satisfaction with social security represents recognition of government services.

Some international literature proposed that satisfactory social security services can alleviate these negative influences (e.g., depression and life stress) caused by social problems to improve citizens’ SWB (Han and Gao, 2020). Retirement may increase the threat of depression for Europeans, Americans, and Turks; however, this risk was likely to be reduced through the Social Security pension. Satisfactory welfare funds could relieve the post-retirement economic stress, which made them happy enough (Kapteyn et al., 2013; Altun and Yazici, 2015). Enjoying benefits from the public pension scheme generally enhanced Chinese life satisfaction, such as Government and Institution Pension, Enterprise Employee Basic Pension, Livelihood Guarantee program (*Dibao*), and Urban–Rural Social Pension Scheme (Gao and Zhai, 2017; Abruquah et al., 2019; Han and Gao, 2020). Pressures to seek medical aid could be mitigated as well as physical health could be in good condition owing to medical insurance coverage, resulting in greater life wellbeing (Tran et al., 2016). Given previous conclusions from the relationship between specific social security programs and SWB, we propose hypothesis 1 as follows from the perspective of holistic satisfaction.

*H1:* Satisfaction with social security may have a positive effect on people’s SWB, namely, people with a higher level of social security satisfaction are likely to have a higher level of SWB.

### Social Security Satisfaction, Social Fairness, and People’s SWB

Social fairness, which encompasses justice and equality, is based on equality but is not synonymous (Chen et al., 2019b). Justice judgment theory assumes that individuals’ perception of fairness is from multiple rules rather than a single rule, including rules of procedure and distribution. Distribution fairness is defined as a person’s belief that is appropriate for rewards, penalties, or resources to be distributed concerning certain criteria. Procedure fairness is defined as an individual’s belief that allocative procedures meeting certain criteria are fair (Leventhal, 1980). As Amartya Sen said, the root of poverty and social inequality is the loss of viability, namely, the unequal distribution of rights and benefits (Sen, 1993, 1999). They will perceive society to be unfair and injustice when people attain unsatisfactory distribution results. Indeed, the essence of social security is to achieve fairness through redistribution. The accessibility and applicability of social security are key to upholding social fairness and justice. In particular, the support for disadvantaged groups demonstrates social fairness and justice, which benefits the enhancement of the entire SWB (Han and Gao, 2020; Wang and Li, 2020).

From the viewpoint of social fairness and justice, we can fully understand the effect of social factors on human behaviors and psychology (Schneider, 2015). Social unfairness will stimulate people to engage in a range of resistant behaviors and negative psychology, which may decline SWB (Ngamaba et al., 2018). Many argued that unfair social distribution and income inequality posed great threats to personal interests, political stability, and social progress, all of which severely decreased people’s happiness and satisfaction, while social fairness was of great benefit to individual happiness (Huang, 2019; Di Martino and Prilleltensky, 2020; Roh, 2021). Wang and Li (2020) found, for example, that disaster-affected people had a higher fairness perception of government relief policy resulting in life satisfaction of post-disasters if they got satisfactory social security. It is concluded that social security satisfaction may have a positive impact on the realization of social fairness, thereby improving SWB. However, relevant research demonstrated that residents’ general perception of fairness is somewhat lower than the socially recognized level (Gao and Qin, 2018). In other words, people are still troubled by a paucity of fairness in life satisfaction. Can governments heighten social fairness by increasing social security satisfaction, which indirectly enhances people’s SWB? Investigations on this indirect relationship are still relatively rare. Thus, we formulate hypothesis 2 in the Chinese context.

*H2:* Social fairness may play a partial mediating role in the relationship between social security satisfaction and people’s SWB.

### Social Security Satisfaction, Social Trust, and People’s SWB

Social trust refers to subjective confidence in institutions or individuals, that is, special trust quantified by institutional trust and generalized trust measured in accordance with interpersonal trust (Zmerli and Castillo, 2015; Zhao and Yu, 2017). The study with cross-national data from 29 Asian countries found that social trust still needed to be improved. High social trust made up only 37%, while low social trust was as high as 54% (Tokuda et al., 2010). In terms of societal theories, social trust is influenced by social environmental factors, it arises in a more equitable social environment. Research in European welfare states showed that welfare security policies and social trust were closely related. A fine welfare system played a role in social protection, which could increase people’s institutional trust and interpersonal trust with reducing unfairness (Hadis, 2014; Daniele and Geys, 2015). Thence, social trust likely thrives in a satisfactory environment of social security.

Social trust is also an indicator that reflects the scenario of social development and contributes to personal life satisfaction (Rothstein and Uslaner, 2005; Helliwell et al., 2016; Appau et al., 2019). Some scholars discovered that social trust, from the perspective of social capital, is significantly associated with people’s SWB (Clark and Lisowski, 2018). For example, Piumatti et al. (2018), drawing from 4,406 Italian samples, also found that social trust had a significant positive effect on life satisfaction. Suriyanrattakorn and Chang (2021) found, using a cross-country panel data set for 97 countries in the period 2011–2019, not only the positive direct relationship between institution trust and life satisfaction, but also the indirect positive effect between them. But a lack of social trust would contribute to financial recession, conflict and contradiction, and poor wellbeing. It is speculated that satisfactory social security policies can strengthen social trust and thereby promote SWB (Kapteyn et al., 2013; Wang and Li, 2020). Churchill and Mishra (2017) found, however, that the effects of trust on SWB are relatively weaker compared to income effects in developing countries. This is further worth verifying in China. We therefore propose hypothesis 3 based on the above-mentioned arguments and conclusions.

*H3:* Social trust may play a partial mediating role in the relationship between social security satisfaction and people’s SWB.

### Social Fairness and Social Trust

Fairness and trust are indispensable considerations for social development; they can complement each other to build an orderly and harmonious society. According to the theory of fairness heuristic, when the overall perception of fairness is formed, people will consider it insightful information to guide and illustrate the relevant fairness information that they may come across later. This fairness will later affect people’s attitudes, emotions, and behaviors towards the outside (Lind, 2001; Jones and Martens, 2009). In this way, the sense of fairness is used as a trust inspiration. The relationship between social fairness and social trust has been noted in the early literature. They believed that fairness is the foundation of trust. In other words, trust arises when social fairness is felt (Brockner, 1996; Brugman et al., 2016).

On the one hand, social fairness perception can significantly increase confidence in government, but unequal social distribution may lead to the government losing trust in the people. The deterioration in social trust is likely to put the entire country in a vicious circle hindering social progress (You, 2012; Zhao and Yu, 2017; Dierckx et al., 2021). On the other hand, social fairness also contributes to interpersonal trust. Studies found that fairness outcomes and resource allocations could reinforce the sense of social belonging (Valcke et al., 2020). Not only did it stimulate personal talents and motivation, but it also improved social acceptance and social trust perception to contribute to social development. Social justice had a statistically positive effect on social trust, regardless of procedural or distributive justice (Gobena and Van Dijke, 2017; Ziller, 2017; Sharma and Yadav, 2018; Zhang and Zhou, 2018). However, there are few domestic studies on the direct relationship between social fairness and social trust. We therefore give hypothesis 4 based on the above theoretical content.

*H4:* Social fairness may positively influence social trust, that is, the higher level of social fairness is connected with the higher degree of social trust.

### Social Security Satisfaction, Social Fairness, Social Trust, and People’s SWB

The above evidences suggested that there is a certain relationship between social security satisfaction, social fairness, social trust, and SWB (Brugman et al., 2016; Helliwell et al., 2016; Appau et al., 2019; Huang, 2019; Di Martino and Prilleltensky, 2020). As social system theory stated, social systems are inherently complex, and the various subsystems of society are interrelated and co-work to achieve the goal of social integration (Luhmann, 1984; Walby, 2007). The material and spiritual content in the society constitutes a stable social structure, which is of great significance to human life (Parsons, 2013).

**Figure 1:**
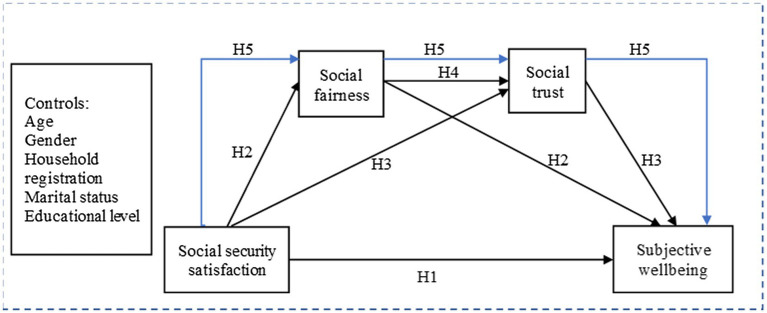
Hypothesized model. SWB, subjective wellbeing.

Actually, social security, social fairness, and social trust are the tangible or intangible components of the social system, impacting people’s life. And the quality of social security system also depends on the level of government governance (Abbott et al., 2015). Therefore, satisfactory social security makes individuals feel that rights and benefits are distributed fairly (Wang and Li, 2020; Wolfson, 2021), likely increasing institutional trust and interpersonal trust (Zhao and Yu, 2017; Ziller, 2017). Some researchers have found that social trust positively affected life satisfaction in the social quality model (Abbott et al., 2015). The results elucidated why economic prosperity is not the only indicator of advancement in life satisfaction, but rather many social determinants must be considered, such as social trust and social inequality. From this, it can be inferred that there is a chain-like relationship between social security satisfaction, social fairness, social trust, and SWB. However, a large number of studies have examined direct paths between them or simple intermediary models, but the supplements of multiple complex models have yet been insufficient. Whether this relationship holds in the Chinese context is unclear. We thus formulate hypothesis 5 and draw a comprehensive hypothetical model framework (see Figure 1).

*H5:* Social security satisfaction may indirectly affect people’s SWB through the serial mediation effect of social fairness and social trust.

## Methods and Measures

### Sample and Data

Chinese Social Survey (CSS) is a large-scale biennial longitudinal sample survey project of the country initiated in 2005 by the Institute of Sociology of the Chinese Academy of Social Sciences. A multistage, stratified, probability-to-size proportional cluster sampling method was employed, and data collection used face-to-face household interviews with a structured questionnaire. The CSS questionnaire was divided into three parts: basic module, replacement module, and hotspot module. It guaranteed the scientific rigor of the survey at multiple levels to obtain high-quality data. During the sampling, this survey designed the sampling frame according to the zoning data of the 5th and the 6th census in China. The survey area contained 31 provinces across the country, including 151 districts, counties, municipalities, and 604 villages or neighborhood committees. More than 7,000 to 10,000 families were visited in each wave. In the execution phase, CSS relied on universities and research institutions all over the country to establish a local investigation team. During the quality control, a certain proportion of questionnaires were reviewed at each survey site to ensure the quality of the questionnaires, and all the questionnaires were double-entered. In addition, the project team anonymized the data so that no respondent was negatively affected by participating in the survey. The research results can infer the status of households aged from 18 to 69 in China.

Based on the requirements of timeliness, adaptability, and sample size, the data in 2017 and 2019 CSS were extracted. These two waves of data in 2017 and 2019 are the latest published data to reflect the current situation. And compared with other waves, they contain consistent survey data that is responsive to the present research. We considered 2017CSS and 2019CSS as the independent samples. After deleting missing values and invalid questionnaires, there were 5,398 data in 2017CSS and 2,580 data in 2019CSS. Then, due to the measurement variables, we pooled the samples together to build a new complete dataset (*N* = 7,978) for quantitative analysis.

### Measures

#### Dependent Variable

SWB, the dependent variable in this study, was reliably reflected in life satisfaction (Larsen et al., 1985; Diener et al., 1997). And self-rated satisfaction with life and income has been used in the SWB measurements (Ng et al., 2017). Hence, we comprehensively adopted the existing measurement dimensions as observed variables. It measured by the question in 2017 and 2019CSS. “Please use 1–10 points to express your satisfaction assessment?” The 10-point Likert scale ranging from 1 (very dissatisfied) to 10 (very satisfied) included, respectively, family financial situation, the environmental situation of residence, and your general satisfaction with life. The total score ranged from 3 to 30. Higher means were associated with higher levels of SWB. These measures had the Cronbach’s alpha coefficient of 0.767.

#### Independent Variable

Social security satisfaction was the independent variable. It referred to the citizens’ rating of social security services that guarantee the basic life. And social security involving pension, education, medical care, employment, housing, etc., has been widely studied in China (Ding, 2017; Ng et al., 2017; Mao and Han, 2018; Tang et al., 2018; Han and Gao, 2020). Therefore, the variable was measured by the following question in this data. “Please use 1–10 points to express your evaluation of the current social security situation?” And the 10-point Likert scale with six items covered pension security, medical security, employment security, urban and rural minimum living security (*Dibao*), basic housing security such as affordable housing, low-rent housing, and public rental housing, and in general, the social security status (“1 = very dissatisfied” to “10 = very satisfied”). The sum was scored from 6 to 60. And higher mean scores of them denoted higher levels of social security satisfaction. These measures had highly internal consistency reliabilities in this study (Cronbach’s *α* = 0.912).

#### Mediating Variables

Social fairness and social trust were mediators in this study. Social fairness meant a subjective assessment of social justice status. Participants responded to the question “What do you think is the level of fairness in the following aspects of current social life?,” regarding public medical care, wealth and income distribution, employment opportunities, pensions and other social security benefits, and rights and benefits between urban and rural areas, with a 4-point Likert scale (“1 = very unfair” to “4 = very fair”). A range of the total score was from 5 to 20. Higher means of 5 items indicated higher degrees of social fairness (Cronbach’s *α* = 0.808). The analogous measurement has been utilized by Zhu et al. (2020).

In addition, social trust was composed of interpersonal trust and institutional trust. It was measured by the following questions in this literature according to previous papers (Zmerli and Castillo, 2015; Zhao and Yu, 2017; Li and Cui, 2018). “Please use 1–10 points to express your evaluation of the trust between people today?” Reassign 1 = very distrust (1–3 points), 2 = distrust (4–5 points), 3 = trust (6–7 points), 4 = very trust (8–10 points). “Do you trust the following agencies? Including central government, district/county government, township government with options from 1 to 4, respectively, indicating “very distrust” to “very trust.” The total value of 4 items ranged from 4 to 16. And higher average values represented a higher level of social trust overall (Cronbach’s *α* = 0.912).

#### Control Variables

Referring to the prior literature, sociodemographic variables can influence the SWB somewhat, thus, we selected determinants as controls (Agrawal et al., 2011; Kapteyn et al., 2015; Abruquah et al., 2019) and recoded them in this research, including age (survey time minus birth year), gender (0 = female, 1 = male), household registration (0 = agricultural residence, 1 = non-agricultural residence, also including resident accounts and other), marital status (0 = married, including first marriage with a spouse and remarriage with a spouse, 1 = unmarried, including cohabitation, divorce, and widows), educational level (0 = uneducated, 1 = primary school, 2 = junior middle school, 3 = high school, 4 = secondary school/technical school, and 5 = college above and others). The detailed measurements are shown in Table 1.

**Table 1:** The details of variable measurements (*N* = 7,978).

Variables	Definitions	Min	Max
Age	The birth year of the respondent. (survey time minus birth year)		
Gender	Gender of the respondent. “0 = female; 1 = male”	0	1
Household registration	Household registration of the respondent. “0 = agricultural residence; 1 = non-agricultural residence, also including resident accounts and other”	0	1
Marital status	Marital status of the respondent.” 0 = married, including first marriage with a spouse and remarriage with a spouse; 1 = unmarried, including cohabitation, divorce and widows”	0	1
Educational level	Educational level of the respondent. “0 = uneducated; 1 = primary school; 2 = junior middle school; 3 = high school; 4 = secondary school/technical school; 5 = college above and others”	0	5
Subjective wellbeing (SWB)	1. Satisfaction with family financial situation. (from “1 = very dissatisfied” to “10 = very satisfied”)	1	10
	2. Satisfaction with the environmental situation of residence. (from “1 = very dissatisfied” to “10 = very satisfied”)	1	10
	3. Satisfaction with your general satisfaction with life. (from “1 = very dissatisfied” to “10 = very satisfied”)	1	10
Social security satisfaction	1. Satisfaction with pension security. (from “1 = very dissatisfied” to “10 = very satisfied”)	1	10
	2. Satisfaction with medical security. (from “1 = very dissatisfied” to “10 = very satisfied”)	1	10
	3. Satisfaction with employment security. (from “1 = very dissatisfied” to “10 = very satisfied”)	1	10
	4. Satisfaction with urban and rural minimum living security (*Dibao*). (from “1 = very dissatisfied” to “10 = very satisfied”)	1	10
	5. Satisfaction with basic housing security such as affordable housing, low-rent housing, and public rental housing. (from “1 = very dissatisfied” to “10 = very satisfied”)	1	10
	6. Satisfaction with the social security status overall. (from “1 = very dissatisfied” to “10 = very satisfied”)	1	10
Social fairness	1. Fairness of public medical care. (from “1 = very unfair” to “4 = very fair”)	1	4
	2. Fairness of wealth and income distribution. (from “1 = very unfair” to “4 = very fair”)	1	4
	3. Fairness of employment opportunities. (from “1 = very unfair” to “4 = very fair”)	1	4
	4. Fairness of pensions and other social security benefits. (from “1 = very unfair” to “4 = very fair”)	1	4
	5. Fairness of rights and benefits between urban and rural areas. (from “1 = very unfair” to “4 = very fair”)	1	4
Social trust	1. Interpersonal trust. (from “1 = very distrust” to “4 = very trust”)	1	4
	2. Trust central government. (from “1 = very distrust” to “4 = very trust”)	1	4
	3. Trust district/county government. (from “1 = very distrust” to “4 = very trust”)	1	4
	4. Trust township government. (from “1 = very distrust” to “4 = very trust”)	1	4

### Analysis

Software AMOS24.0 and SPSS26.0 and Macro programs (PROCESS v3.50) written by Hayes (2017) were employed in the present study (*N* = 7,978).

We first used the SPSS26.0 to perform reliability tests, descriptive analysis, correlations. Cronbach’s alpha is the most extensively applied objective measure of reliability when multiple-item measures of a concept or construct are implemented (Tavakol and Dennick, 2011). Cronbach’s test in the SPSS26.0 runs the reliability analysis of key variables. And the acceptable values of alpha are more than 0.7 in most reports (Bland and Altman, 1997; Tavakol and Dennick, 2011), which was also adopted in this study. Composite reliability (CR) and average variance extracted (AVE) verified the constructs’ reliability and convergent validity. A general guideline is that CR values and AVE values should be higher than 0.7 and 0.4, respectively (Netemeyer et al., 2003; Hair et al., 2009).

Descriptive statistics is the fundamental step for discovering central and discrete trends in data. This process was run in the SPSS26.0 to get frequencies, percentages, means, and standard deviation (SD) among variables. Correlation analysis is a crucial tool to understand the basis vectors between two quantities, and it has a close relationship with regression analysis (Cohen et al., 1983; Lindley, 1990). When there is a high correlation between variables, it makes sense to perform regression analysis to find a specific form of correlation. The correlation results of all variables were achieved by Pearson correlation analysis in this software so that we can further analyze the correlated form between all variables.

Then, confirmatory factor analysis (CFA) was performed by AMOS24.0 to check the model fit. CFA is a common method to test the relationship between latent variables and observed variables. We drew the confirmatory model, including SWB, social security satisfaction, social fairness, and social trust, and run it. The results of Chi-square (*χ*^2^), Comparative Fit Index (CFI), and Root Mean Square Error of Approximation (RMSEA) were mainly used to measure the model fit. Generally, smaller *χ*^2^ (*p* > 0.05) is better, but larger samples can be considered separately. CFI is as close as possible to 1, if CFI >0.9, it is very satisfying. If RMSEA <0.08, it indicates an acceptable fit of the model with the degrees of freedom (Bollen, 1989; Bentler, 1990; Browne and Cudeck, 1992).

Finally, Model 6 in PROCESS3.50 was employed to test this serial mediation model. In this module, there was one dependent variable (SWB) and one independent variable (social security satisfaction), with two mediators (social fairness and social trust) operating in serial and several controls. The confidence level for all confidence intervals (CI) in output was 95%. The number of bootstrap samples for percentile bootstrap CI was 5,000. As suggested by Preacher and Hayes (2008), the effect is significant if CI does not include zero. We took social security satisfaction as an independent variable with social fairness, social trust, and SWB as dependent variables in three regression models, respectively. Hypothesis testing was performed based on the regression results of three models in turn. In the study, regression analysis equations were as follows.


(1) $$M_{SF}=\beta_{MSF}+\eta_{1}X_{SS}+\xi_{MSF}$$

(2) $$M_{ST}=\beta_{MST}+\eta_{2}X_{SS}+\eta_{3}M_{SF}+\xi_{MST}$$

(3) $$Y_{SWB}=\beta_{YSWB}+\mu_{1}X_{SS}+\gamma_{1}M_{SF}+\gamma_{2}M_{ST}+\xi_{SWB}$$

where SF was social fairness, ST was social trust, SS was social security satisfaction, SWB was subjective wellbeing, *ξ* was control variables, including age, gender, household accounts, marital status, and educational level in the study. *β* was a constant. *η*, *μ*, *γ* were regression coefficients. And the direct effect of SS on SWB was estimated by *μ*_1_ in equation (3). The indirect effect of SS on SWB through SF, ST, and SF and ST in serial, respectively, was *η*_1_*γ*_1_, *η*_2_*γ*_2_, and *η*_1_*η*_3_*γ*_2_. The total indirect effect of SS on SWB was the sum of *η*_1_*γ*_1_, *η*_2_*γ*_2_ and *η*_1_*η*_3_*γ*_2_. When added to the direct effect (*μ*_1_), the result was the total effect of SS on SWB.

## Results

### Descriptive Results and Correlations

The distribution of demographic characteristics in the descriptive results was relatively balanced overall. In accordance with age, the number of 35 to 54 years old was the highest at 43.5%, the average age was 43 (SD = 14.41). The gender distribution was relatively proportional to men and women at 46.5 and 53.5%, respectively. And agricultural households accounted for the majority (65.5%), while non-agricultural households accounted for 34.5%. According to marital status, 76.5% of the persons were married, 23.5% were unmarried, including cohabitation, divorce, and widows. In terms of the educational level, most people had a junior middle school diploma (31.1%) or a college degree and above (23.1%), and only 7% of people are uneducated. Full information is given in Table 2.

**Table 2:** Sociodemographic characteristics (*N* = 7,978).

		Total		2017		2019	
		*N*	**%**	*N*	**%**	*N*	%
Survey time	2017	5,398	67.7				
	2019	2,580	32.3				
Age (M = 43, SD = 14.41)	≤34 years old	2,545	31.9	1,667	30.9	878	34.0
	35–54 years old	3,472	43.5	2,347	43.5	1,125	43.6
	≥55 years old	1,961	24.6	1,384	25.6	577	22.4
Gender	Female	4,269	53.5	2,871	53.2	1,398	54.2
	Male	3,709	46.5	2,527	46.8	1,182	45.8
Household registration	Agricultural residence	5,223	65.5	3,501	64.9	1722	66.7
	Non-agricultural residence	2,755	34.5	1,897	35.1	858	33.3
Marital status	Married	6,104	76.5	4,179	77.4	1925	74.6
	Unmarried	1,874	23.5	1,219	22.6	655	25.4
Educational level	Uneducated	556	7	413	7.7	143	5.5
	Primary school	1,499	18.8	1,058	19.6	441	17.1
	Junior middle school	2,481	31.1	1,690	31.3	791	30.7
	High school	1,103	13.8	759	14.1	344	13.3
	Secondary school/technical school	494	6.2	322	6.0	172	6.7
	College above and others	1,845	23.1	1,156	21.4	689	26.7

*N, frequency; %, percent; M, mean; SD, standard deviation*.

**Figure 2:**
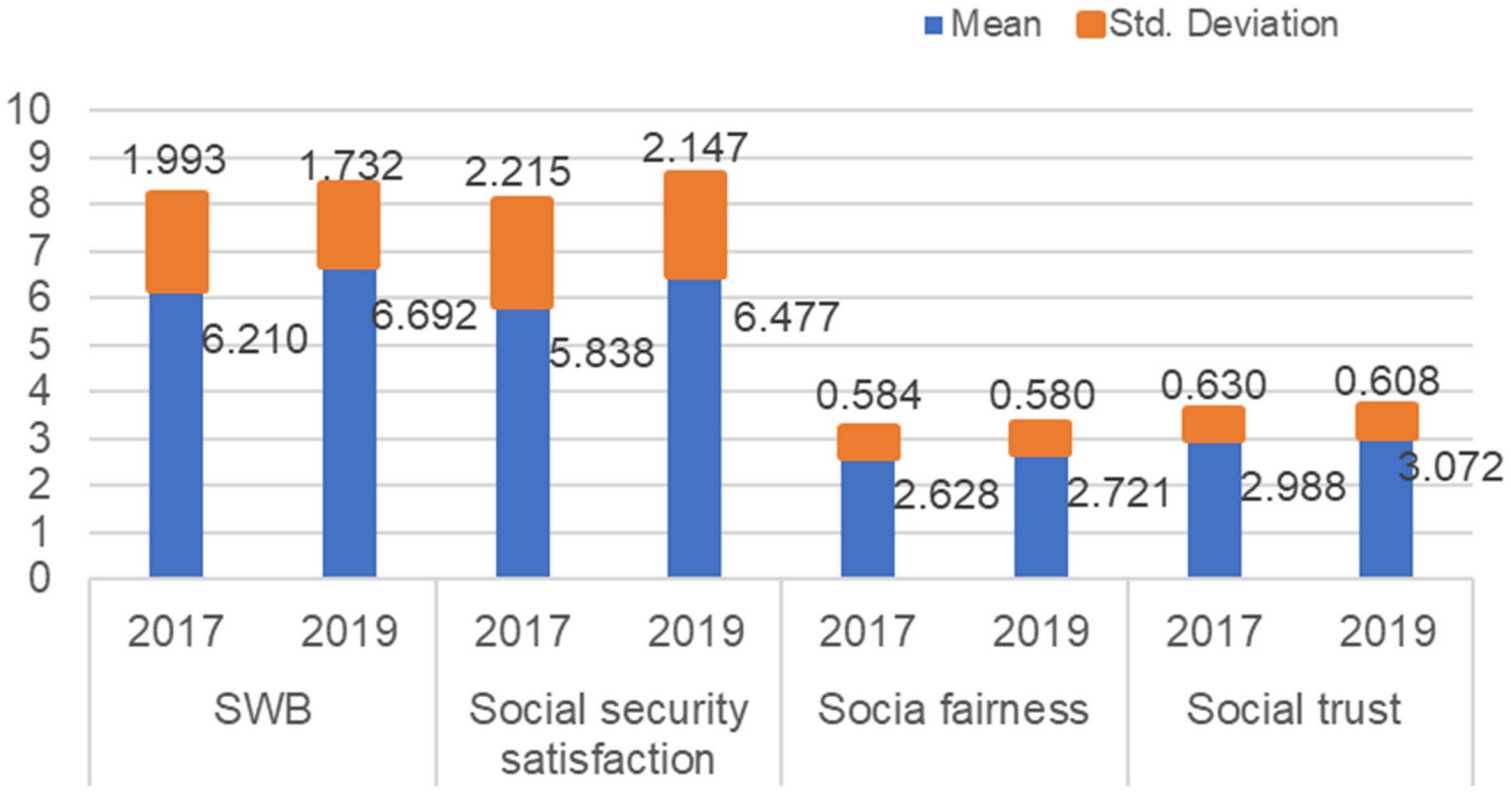
The mean trend of the core variables in 2017 and 2019 (*N* = 7,978). SWB, subjective wellbeing; SS, social security satisfaction; SF, social fairness; ST, social trust.

Descriptive statistics of research variables in the study can be found in Table 3. The means of SWB was 6.37 (SD = 1.93), indicating that the SWB level was generally high, but there was a certain discrepancy. The actual means of social security satisfaction (M = 6.04, SD = 2.21), social fairness (M = 2.66, SD = 0.58), and social trust (M = 3.02, SD = 0.62) were higher than the theoretical means. It suggested that the overall social security satisfaction, fairness, and trust were in good conditions, but there were certainly individual differences. Specifically, the levels of people’s SWB, social security satisfaction, social fairness, and social trust kept all ascending from 2017 to 2019, and the gap was gradually narrowing (see Figure 2). The average values of SWB increased from 6.21 to 6.692, the SD decreased by 0.261. The means of people’s satisfaction with social security increased by 0.639, and the SD decreased from 2.215 in 2017 to 2.147 in 2019. The mean values of social fairness (2.628 to 2.721) and social trust (2.988 to 3.072) increased slightly, but the differences had not changed obviously.

**Table 3:** Correlations and descriptive statistics of the study variables (*N* = 7,978).

	M	SD	1	2	3	4	5	6	7	8	9
1	6.37	1.93	1								
2	6.04	2.21	0.550^**^	1							
3	2.66	0.58	0.331^**^	0.493^**^	1						
4	3.02	0.62	0.359^**^	0.501^**^	0.475^**^	1					
5	43	14.41	−0.084^**^	0.023^*^	0.041^**^	0.120^**^	1				
6	0.46	0.50	0.023^*^	0.022	−0.007	0.026^*^	0.050^**^	1			
7	0.35	0.48	0.091^**^	0.055^**^	−0.042^**^	0.007	−0.019	0.011	1		
8	0.23	0.42	0.061^**^	0.055^**^	0.034^**^	0.015	−0.414^**^	0.088^**^	0.049^**^	1	
9	2.63	1.59	0.194^**^	0.086^**^	−0.045^**^	−0.013	−0.513^**^	0.086^**^	0.423^**^	0.287^**^	1

1, subjective wellbeing; 2, social security satisfaction; 3, social fairness; 4, social trust; 5, age; 6, gender; 7, household registration; 8, marital status; 9, educational level; M, mean; SD, standard deviation. ^**^p < 0.01, ^*^p < 0.05 (2-tailed).

From the correlation results (see Table 3), satisfaction with social security, social fairness, and social trust all correlated significantly positively with SWB. Compared with social fairness (*r* = 0.331, *p* < 0.01) and social trust (*r* = 0.359, *p* < 0.01), the positive correlation between social security satisfaction and SWB was stronger (*r* = 0.55, *p* < 0.01). Secondly, social security satisfaction had a positive correlation with social fairness and social trust, and the correlation coefficients were 0.493 (*p* < 0.01) and 0.501 (*p* < 0.01), respectively. In addition, social fairness and social trust were also significantly positively correlated (*r* = 0.475, *p* < 0.01). It was meaningful to perform further regression analysis to test the specific interaction form between variables.

### Confirmatory Factor Analysis and Reliability Test

**Figure 3:**
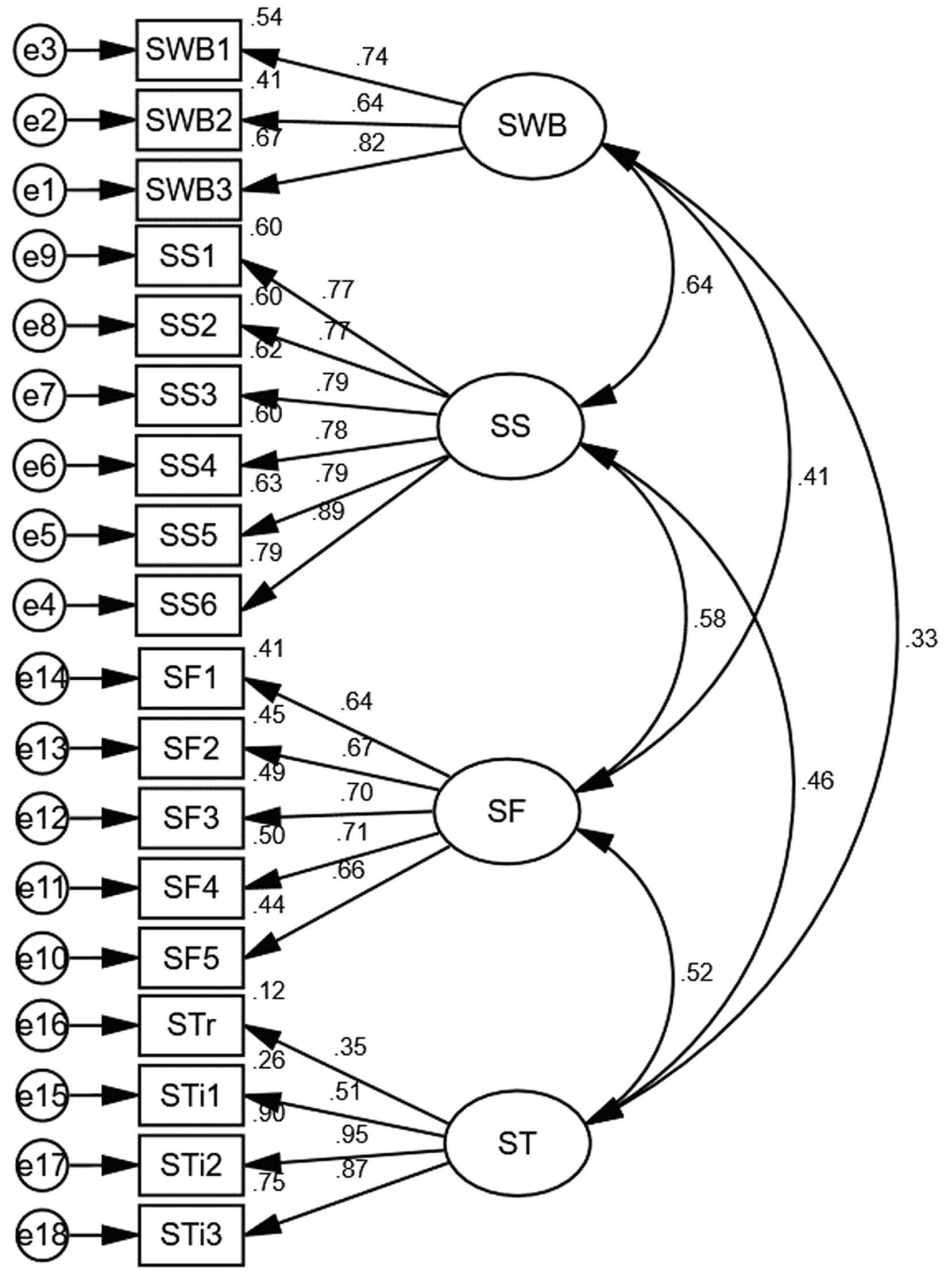
The standardized results of CFA (*N* = 7,978). SWB, subjective wellbeing.

The CFA results showed that *χ*^2^ = 5177.61, df = 129 (*p* < 0.001). RMSEA = 0.07 (RMSEA <0.08). CFI = 0.93 (CFI >0.9). Taking into account the large sample size (*N* = 7,978) and model complexity, however, even if *χ*^2^ is significant and large, models with higher satisfaction with other values can be accepted (Bollen, 1989; Bentler, 1990; Browne and Cudeck, 1992). The Cronbach’s *α* >0.7, AVE >0.4, CR >0.7 in the variables, and standardized loading factor values were above 0.3. All of them were within the acceptable range (Agnew, 1991; Bland and Altman, 1997; Netemeyer et al., 2003; Hair et al., 2009; Tavakol and Dennick, 2011). Therefore, the measurement model was satisfactory and reliable. Latent variables can be reflected by observed variables in this research (see Table 4; Figure 3).

**Table 4:** Standardized loading factors of observed variables on latent construct and reliability and validity (*N* = 7,978).

Latent variable	Observed variable	Standardized loading factor	Cronbach’s *α*	AVE	CR
SWB	SWB1 your satisfaction with family financial status	0.736	0.767	0.540	0.777
	SWB2 your satisfaction with the environmental situation of residence	0.639			
	SWB3 overall your satisfaction with life	0.819			
Social security satisfaction	SS1 pension security	0.773	0.912	0.639	0.914
	SS2 medical security	0.775			
	SS3 employment security	0.785			
	SS4 *Dibao*	0.778			
	SS5 basic housing security	0.791			
	SS6 in general, the social security status	0.887			
Social fairness	SF1 public medical care	0.644	0.808	0.459	0.809
	SF2 employment opportunities	0.669			
	SF3 wealth and income distribution	0.703			
	SF4 pensions and other social security benefits	0.71			
	SF5 rights and benefits between urban and rural areas	0.66			
Social trust	STr interpersonal trust	0.347	0.733	0.508	0.784
	STi1 central government	0.51			
	STi2 district/county government	0.947			
	STi3 township government	0.868			

*SWB, subjective wellbeing*.

### Test of Regression Model

According to the regression results in Table 5 (*N* = 7,978), social security satisfaction, respectively, had a significant positive effect on social fairness, social trust, and people’s SWB. And all hypotheses were supported.

**Table 5:** Regression coefficient and significance of the serial mediation model (*N* = 7,978).

	Model 1 (Outcomes: SF)				Model 2 (Outcomes: ST)				Model 3 (Outcomes: SWB)			
	B (β)	SE	t	95%-BootLLCI, BootULCI	B (β)	SE	t	95%-BootLLCI, BootULCI	B (β)	SE	t	95%- BootLLCI, BootULCI
Age	0.000	0.001	0.218	[−0.001, 0.001]	0.006	0.001	10.796^***^	[0.005, 0.007]	−0.007	0.002	−4.448^***^	[−0.010, −0.004]
	(0.003)				(0.127)				(−0.053)			
Gender	−0.016	0.012	−1.346	[−0.039, 0.006]	0.011	0.012	0.920	[−0.011, 0.032]	0.015	0.036	0.421	[−0.054, 0.083]
	(−0.013)				(0.009)				(0.004)			
Household registration	−0.047	0.014	−3.427^**^	[−0.073, −0.019]	−0.016	0.014	−1.156	[−0.034, 0.017]	0.045	0.042	1.065	[−0.039, 0.124]
	(−0.038)				(−0.012)				(0.011)			
Marital status	0.045	0.015	3.043^**^	[0.016, 0.075]	0.043	0.015	2.884^**^	[0.020, 0.075]	−0.135	0.046	−2.928^**^	[−0.232, −0.042]
	(0.033)				(0.029)				(−0.030)			
Educational level	−0.029	0.005	−6.026^***^	[−0.038, −0.020]	0.013	0.005	2.579^*^	[0.001, 0.020]	0.164	0.015	11.033^***^	[0.136, 0.194]
	(−0.079)				(0.032)				(0.136)			
*SS*	0.132	0.003	51.289^***^	[0.127, 0.138]	0.098	0.003	32.597^***^	[0.081, 0.093]	0.395	0.010	40.311^***^	[0.377, 0.423]
	(0.501)				(0.347)				(0.454)			
*SF*					0.319	0.011	28.124^***^	[0.284, 0.332]	0.215	0.036	5.918^***^	[0.145, 0.302]
					(0.298)				(0.065)			
*ST*									0.334	0.034	9.732^***^	[0.237, 0.397]
									(0.108)			
*R*	0.504				0.576				0.584			
*R* ^2^	0.254				0.331				0.341			
*F(df)*	451.081 _(6)_^***^				563.748_(7)_^***^				514.27_(8)_^***^			

SWB, subjective wellbeing; SS, social security satisfaction; SF, social fairness; ST, social trust. B, unstandardized regression coefficients; β, standardized regression coefficients; SE, standard error. F, the significance test of the regression equation; t, the significance test value of the regression parameter; R^2^, statistic relates to variation between individuals. BootLLCI, bootstrap lower bound of the confidence interval; BootULCI, bootstrap upper bound of the confidence interval, ^***^p < 0.001, ^**^p < 0.01, ^*^p < 0.05.

In Model 3 with SWB as the outcome, the results illustrated that social security satisfaction had a positive effect on SWB (*β* = 0.454, *p* < 0.001, 95% CI = [0.377, 0.423]). People with a high level of social security satisfaction were likely to have a high level of SWB, supporting H1. 34.1% of the variance in SWB could be explained in this model.

The regression results based on social fairness as an output variable (Model 1) showed that the high-level social security satisfaction was associated with the high-level social fairness (*β* = 0.501, *p* < 0.001, 95% CI = [0.127, 0.138]), and 25.4% of the variance in social fairness in China could be explained. Based on the findings of Model 3, social fairness had a significant positive impact on SWB (*β* = 0.065, *p* < 0.001, 95% CI = [−0.039, 0.124]). It can be seen that social fairness played a partial mediation role in the relationship between social security satisfaction and people’s SWB. H2 was supported, that is, social security satisfaction indirectly influenced people’s SWB through social fairness.

As expected, in Model 2 with social trust as the dependent variable, social security satisfaction positively influenced social trust (*β* = 0.347, *p* < 0.001, 95% CI = [0.081, 0.093]). People with a high level of social security satisfaction were likely to possess a high-level social trust. And Model 3 results also showed that social trust was significantly associated with SWB (*β* = 0.108, *p* < 0.001, 95% CI = [0.237, 0.397]). H3 was supported because of it. The partial mediating role of social trust was played in the relationship between social security satisfaction and people’s SWB.

In Model 2, the findings also demonstrated that social fairness positively influenced social trust (*β* = 0.298, *p* < 0.001, 95% CI = [0.284, 0.332]). It revealed that higher levels of social fairness were correlated with higher levels of social trust, and 33.1% of the variance in social trust could be explained. H4 was supported.

**Figure 4:**
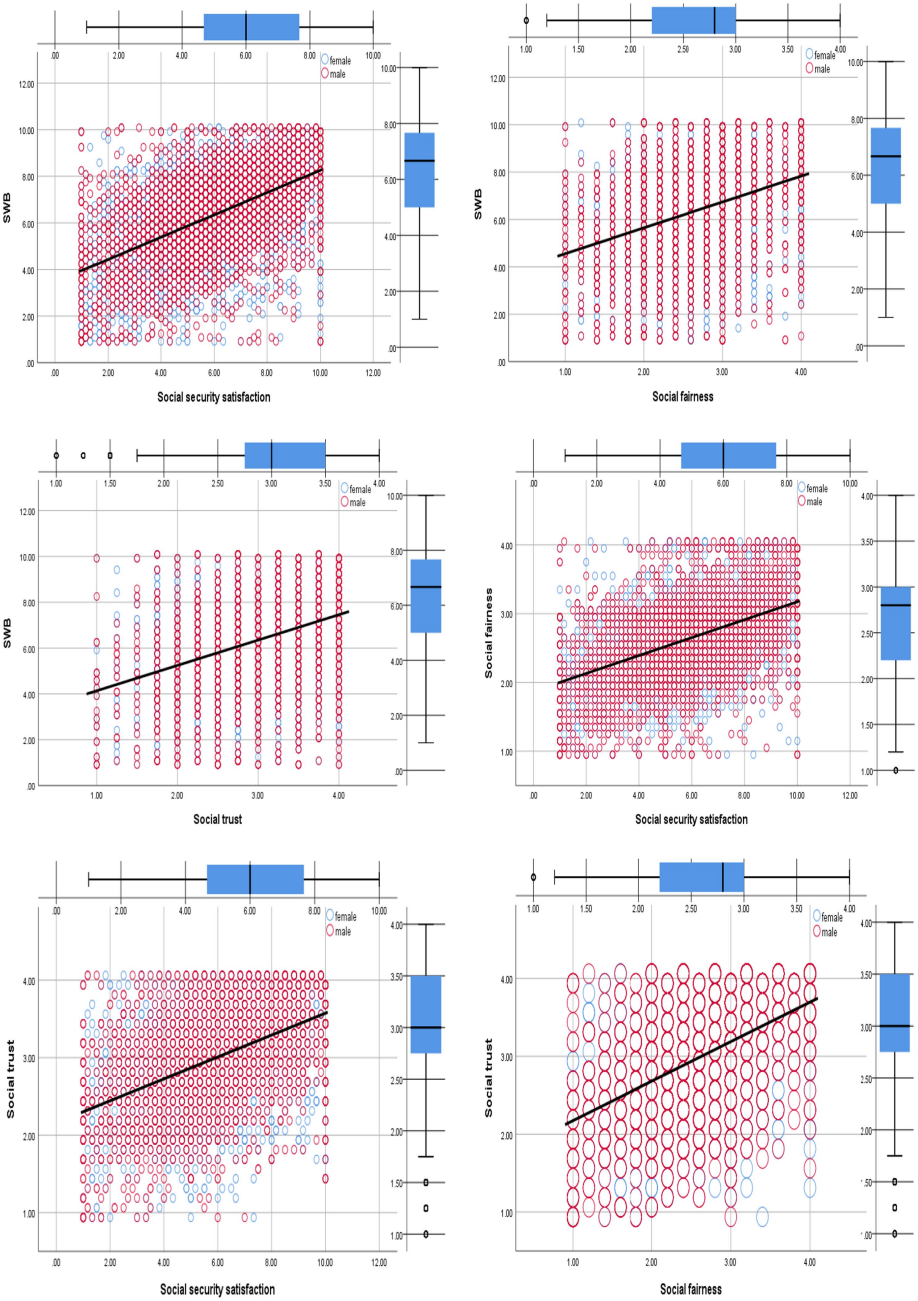
Regression variable graph between social security satisfaction, social trust, social fairness, and SWB colored by gender (*N* = 7,978).

**Figure 5:**
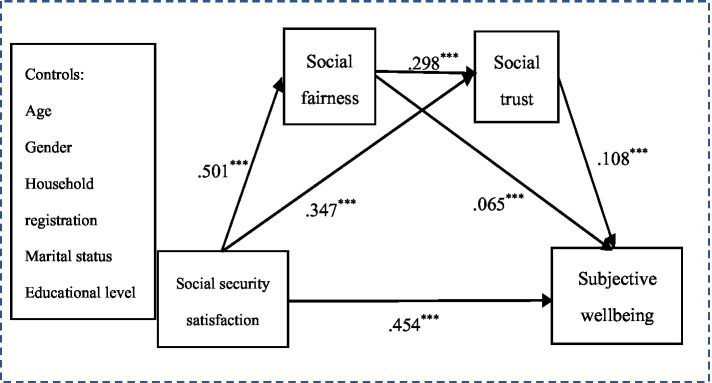
Standardized solutions for the serial mediation model (^***^p < 0.001; *N* = 7,978).

The above regression results indicated that social security satisfaction had a significant indirect effect on people’s SWB through social fairness and social trust. And the serial mediation role of social fairness and social trust was played in the relationship between social security satisfaction and SWB, supporting H5. To observe the relationship between variables more intuitively, we have also drawn the paths and linear regression graphs, showing the overall fitting trend. Details are presented in Figures 4, 5.

The effect results (see Table 6) showed that the total effect of SWB explained was 47% in the serial mediation model. The direct effect of social security satisfaction on SWB was 39.5%, accounting for 83.99% of the total effect, and the indirect effect was 15.99% of the total effect. Specifically, the indirect effect mediated by social trust (43.48%) was greater than that mediated by social fairness (37.9%). The serial intermediary effect with social fairness and social trust accounted for 18.75% of the indirect effect. The bootstrap CI (95%) in all paths did not include zero, indicating that the effects were significant. And this serial mediation model was supported.

**Table 6:** Mediation path effect values and relative mediation effect (*N* = 7,978).

	Path	Effect	BootSE	BootLLCI-95%	BootULCI-95%	RE
Total effect		0.470	0.008	0.455	0.486	
Direct effect	SS- > SWB	0.395	0.010	0.376	0.414	83.99%
Indirect effect		0.075	0.007	0.062	0.088	15.99%
1	SS- > SF- > SWB	0.029	0.006	0.018	0.039	37.90%
2	SS- > ST- > SWB	0.033	0.004	0.025	0.041	43.48%
3	SS- > SF- > ST- > SWB	0.014	0.002	0.011	0.018	18.75%
	1 minus 2	−0.004	0.008	−0.020	0.010	
	1 minus 3	0.014	0.006	0.002	0.026	
	2 minus 3	0.019	0.003	0.014	0.024	

*SWB, subjective wellbeing; SS, social security satisfaction; SF, social fairness; ST, social trust. BootSE, bootstrap standard errors. BootLLCI, bootstrap lower bound of the confidence interval; BootULCI, bootstrap upper bound of the confidence interval; RE, relative effect*.

In addition, among the control variables, age could reversely impact SWB (*β* = −0.053, *p* < 0.001). As age increased, the level of SWB would decrease. Education level could positively influence SWB (*β* = 0.136, *p* < 0.001), namely, a high educational level was associated with a high SWB level. The marital status could significantly influence SWB (*β* = −0.03, *p* < 0.01). The unmarried would be a lower SWB than the married. There was no statistically significant relationship between other controls and SWB (see Table 5).

## Discussion and Conclusion

In summary, this study used data from CSS in 2017 and 2019 to test the relationship among social security satisfaction, social fairness, social trust, and people’s SWB. The results suggested that the actual levels of social security satisfaction, social fairness, social trust, and people’s SWB were higher than the ideal means. The social status was better in 2019 than in 2017. Social security satisfaction had a positive effect on social fairness, social fairness positively influenced social trust, and social security satisfaction indirectly affected people’s SWB through the mediating effect of social fairness and social trust, respectively. The serial mediation effect of social fairness and social trust existed in the relationship between social security satisfaction and SWB. Discussions are as follows.

Firstly, the results supported that social security satisfaction positively influenced people’s SWB, which is in line with prior studies (Altun and Yazici, 2015; Tran et al., 2016; Abruquah et al., 2019). Under the Chinese governance model, people are highly dependent on the government, and they are eager to get more policy supports to release life pressure. Hence, social security plays a prominent role in promoting economic prosperity, maintaining social stability, and improving people’s livelihoods. As a public service, the field and scope of social security coverage have a direct effect on people’s life quality. Especially for vulnerable groups in society, social security, such as medical care, education, employment, pensions, and welfare, is the final obstacle to receiving support. Satisfactory social security ensures a more balanced distribution of national interests and narrows the gap between rich and poor. Under the premise of fulfilling people’s basic lives, people with high-level social security satisfaction are more likely to have a good life quality (Sun and Xiao, 2011; Abbott et al., 2015). This may be the reason for the results.

Secondly, our results showed that social fairness played a partial mediating role between social security satisfaction and people’s SWB. Social security satisfaction indirectly influenced SWB through the mediating effect of social fairness. One reason may be due to the fact that the rapid development of China’s economy has also brought about the wealth gap and the rural–urban duality. Inequality cannot be eliminated by relying on individual strength. The government shoulders a macro-control role for fairness and justice. A tool of governance is the social security system, that is, social security improves overall social fairness by redressing injustices at the beginning and end. Therefore, people’s sense of acquisition and satisfaction with social security strongly influences their assessment of social fairness, and also influences their perception of belonging and identification with society. It can instill their confidence in life benefiting SWB, which consistent with the empirical data derived from 28 EU countries (Di Martino and Prilleltensky, 2020). When people lived in a fair environment, they could get the resources and assistance they deserved to withstand the risks and harms caused by class stratification. And social fairness is able to ignite people’s hope for future development and reduce the risk of depression, which is beneficial for their SWB (Roh, 2021).

Thirdly, we also confirmed that social security satisfaction indirectly affected people’s SWB through the mediating effect of social trust. As societal theory believed, the formation of social trust requires a friendly social environment, survival, and safety are keys. The government help citizens overcome life risks and difficulties by giving them all life guarantees that improve their satisfaction with social security. This will increase people’s trust in the government to build a positive social network system promoting general social trust (Zhao and Yu, 2017). Thence, social security satisfaction has a positive impact on social trust in this text. A confidence environment free of prejudice and discrimination allows people to behave authentically and live more relaxed and happier. That’s why, even in developing China, social trust had a significantly positive impact on SWB. But it disagrees with the previous research (Churchill and Mishra, 2017). Perhaps a high level of social trust is conducive to improving social quality, and people’s SWB can also be influenced indirectly or directly in this way. Although China is not a developed country, the status quo of society is desirable. Social trust is likely to affect curvedly on people’s SWB by improving other factors (Kapteyn et al., 2013; Piumatti et al., 2018). This finding therefore is expected.

Fourthly, the results showed that social fairness had a positive effect on social trust, which supports the relevant literature (Zmerli and Castillo, 2015; Brugman et al., 2016; Zhang and Zhou, 2018). A study in Latin America suggested that distributive unfairness and income inequality are associated with lower levels of political trust (Zmerli and Castillo, 2015). Because procedural fairness is of great importance to the survival of vulnerable groups (Dierckx et al., 2021). Ziller (2017) also found that institutional fairness can ensure the fairness of rights and interests by narrowing the gaps between different groups, which improved social integration and tolerance as well as social trust. Not only will it enlarge people’s willingness to work and engage, but it will also increase political support and acceptance (Gobena and Van Dijke, 2017; Sharma and Yadav, 2018). It also proved the applicability of fairness heuristic theory in the Chinese context. Positive human behaviors are more likely to be inspired in the fair surrounding, such as trust and trustworthiness (Lind, 2001; Jones and Martens, 2009). The result may be because the sense of fairness could lead people to lower their vigilance and treat others equally, it prevents discrimination, prejudice, and conflict in society (Dierckx et al., 2021). This benefits to building a harmonious society and interpersonal trust and institutional trust. Thus, the high degree of social fairness was correlated with the high level of social trust in the present study.

Finally, based on the above conclusions, we verified the serial mediating effect of social fairness and social trust in this model through the regression test. According to social system theory (Luhmann, 1984), the elements and connections of different social systems are interconnected. When an error occurs in a subsystem, other aspects are adversely affected. On the contrary, the improvement of one system function also leads to the optimization of other functions. The coordinated operation of the social system can create a stable social environment that is of positive significance to people’s lives. As an important part of the social governance system, the quality of social security may have a series of social effects. Social security satisfaction plays a central role in people’s SWB through a chain mechanism in the social system. To be more specific, satisfactory social security can associate with great social fairness, thereby strengthening social trust, forming benign serial effects, and then positively influencing people’s SWB. This is a possible reason why this serial mediation model was supported.

Additionally, the data also showed that age, marital status, and educational level were significantly related to SWB. Age was negatively influenced people’s SWB level, namely, people’s SWB declined with aging, which supports the U-shaped relationship between age and wellbeing (Schwandt, 2016). People’s expectations for life will increase as people grow older, and they will lower their expectations until they enter the old stage, and the reality gap will also change from big to small, creating a trend that their life satisfaction first decreases and then increases. Middle-aged Chinese with many life stresses such as divorce, unemployment, daily expenses, etc., have gone through the process of social transformation that has a huge impact on their lives, which may minimize life satisfaction (Sun and Xiao, 2011). The age distribution in this research was concentrated in the middle-aged stage. It was in the first half of the U-shaped relationship transition, so this result can be accepted. The results indicated that married people had higher SWB than unmarried, which is consistent with the literature (Lawrence et al., 2019). Intimacy is a critical social relationship for people to accept emotional support, and being able to share the life stress will undoubtedly increase married people’s SWB. However, the relationship between educational levels and SWB failed to support some studies (Sun and Xiao, 2011; Kristoffersen, 2018). Their research showed that there was no obvious relation or negative relationship between educational level and SWB. Because well-educated people placed higher demands on living conditions, they needed to meet expectations at the expense of free time and overloaded work, which was correlated with lower SWB, while our results supported that educational level positively influenced SWB (Jongbloed, 2018), namely, well-educated people had a high level of SWB. This may be because of different research backgrounds. In contemporary Chinese society, educational attainment is closely related to employment opportunities, personal achievements, and welfare benefits. Compared to people with a low education level, people with a high educational degree are more likely to own social status, prestige, and gratifying welfare benefits, all of which contribute to a high level of SWB (Ruiu and Ruiu, 2019).

## Theoretical Implications

This research mainly contributes to three theoretical implications. First of all, our findings enrich the academic achievements and make up for literature gaps. The vast literature focused on the objective delivery of social security but ignored the feelings of beneficiaries. However, as direct recipients of social security benefits, citizens’ satisfaction with social security is a powerful measure of the quality of social security. We examined the relationship at the subjective view of citizens to offset the objective rigidity. The results more realistically reflected how people feel. It adds new conclusions to the academic research.

Second, this study formulated a serial mediation model that can provide a reference for the relative research. Prior studies paid attention to the effect of individual factors or simple intermediary models on SWB. This leaves the research lacking a certain integrity. But our empirical model showed that social security satisfaction can influence indirectly SWB through the serial mediation model of social fairness and social trust. It notes that we should view and address social problems from a networked but not separated perspective to sidestep a vicious circle. Variables in this model can be replaced by other variables to carry out corresponding studies.

Third, it also proved the suitability of some foreign theories in the Chinese context. We give hypotheses in terms of theories, such as Maslow’s need hierarchy theory, societal theories, fairness heuristic theory, social system theory, etc., to analyze the rationality of relationships. Unsurprisingly, the theoretical model and empirical results corroborated each other. This also indicates that theories in this research can explain the relationships between social security satisfaction, social fairness and trust, and people’s SWB. In the future, these theories can be further developed and refined to better adapt to localization scenarios.

## Practical Implications

As far as practical implications, we provide targeted policy suggestions for improving people’s SWB. Firstly, the empirical results illustrated that social security satisfaction can directly impact on people’s SWB. Thus, to protect the interests of direct beneficiaries, democratic participation and policy security should be fully integrated into social governance to pledge social order, the policy applicability. This is a necessary procedure to enhance the levels of social trust and fairness (Keping, 2017). Because democratic engagement can ensure the openness and fairness of welfare systems, achieving fair distribution of benefits, and effective policies are good for social trust, thereby ensuring people’s SWB.

Secondly, because of the partial mediating effect of social fairness and trust, the state should ensure fairness and justice as much as possible in building and improving the social security system. It must be ensured that the interests of high-income groups are not harmed and that low-income groups can receive support, whereby the actual claims of middle-income groups are also taken into account. It is necessary to increase the accessibility and satisfaction of all citizens to social security and lay the foundation of social fairness and trust by expanding social security coverage. This will contribute to the high-level SWB.

Finally, due to the serial mediation effect of social fairness and trust, government of China needs to put in place a perfect monitoring and feedback mechanism to pledge the implementation of social security policy. External coercion is a powerful guarantee to avoid corruption and inefficiency in administrative systems. This also is beneficial for the improvement of social security, increasing satisfaction with fairness and trust. And public opinions need to be heard to improve social security services and enhance people’s SWB achieving the ultimate goal of social governance.

## Limitations

Undoubtedly, there are also certain limitations in this study. First, secondary data may have timeliness issues resulting in potential disputes. In the follow-up, it is necessary to collect first-hand longitudinal data to further test the causal link and improve the model. Second, only the roles of social security satisfaction, fairness, and trust are considered in this study, but other sociodemographic variables such as those shown in the findings have implications for further study. There is still room for improvement in the research scope and issues. Thirdly, we have to do more cross-country studies, because research conclusions in the Chinese context may be of greater significance for China’s development than other countries.

## Data Availability Statement

Publicly available datasets were analyzed in this study. This data can be found here: The raw data that support the findings of this study are openly available on the official website of Chinese Social Quality Data Archive at: http://csqr.cass.cn/DataExplore/, reference number 2017CSS and 2019CSS. And data in this study can be obtained by connecting with the corresponding author.

## Author Contributions

NL wrote and revised the full-text. MH critically revised the manuscript and guided the process. All authors reviewed and approved the final version of the manuscript.

## Conflict of Interest

The authors declare that the research was conducted in the absence of any commercial or financial relationships that could be construed as a potential conflict of interest.

## Publisher’s Note

All claims expressed in this article are solely those of the authors and do not necessarily represent those of their affiliated organizations, or those of the publisher, the editors and the reviewers. Any product that may be evaluated in this article, or claim that may be made by its manufacturer, is not guaranteed or endorsed by the publisher.
